# EphrinB2–EphB4 Signaling in Neurooncological Disease

**DOI:** 10.3390/ijms23031679

**Published:** 2022-01-31

**Authors:** Andras Piffko, Christian Uhl, Peter Vajkoczy, Marcus Czabanka, Thomas Broggini

**Affiliations:** 1 Department of Neurosurgery, University Medical Center Hamburg-Eppendorf, 20251 Hamburg, Germany; a.piffko@uke.de; 2 Department of Radiation and Cellular Oncology, University of Chicago, Chicago, IL 60637, USA; 3 Ludwig Center for Metastasis Research, University of Chicago, Chicago, IL 60637, USA; 4 Department of Neurosurgery, University Medicine Charité, 10117 Berlin, Germany; christian.uhl@charite.de (C.U.); peter.vajkoczy@charite.de (P.V.); 5 Department of Neurosurgery, University Hospital Frankfurt, 60528 Frankfurt am Main, Germany; marcus.czabanka@kgu.de

**Keywords:** EphB4, ephrinB2, metastasis, glioblastoma, glioma, bone, brain metastasis, spinal metastasis, neurooncology, bone metastasis

## Abstract

EphrinB2–EphB4 signaling is critical during embryogenesis for cardiovascular formation and neuronal guidance. Intriguingly, critical expression patterns have been discovered in cancer pathologies over the last two decades. Multiple connections to tumor migration, growth, angiogenesis, apoptosis, and metastasis have been identified in vitro and in vivo. However, the molecular signaling pathways are manifold and signaling of the EphB4 receptor or the ephrinB2 ligand is cancer type specific. Here we explore the impact of these signaling pathways in neurooncological disease, including glioma, brain metastasis, and spinal bone metastasis. We identify potential downstream pathways that mediate cancer suppression or progression and seek to understand it´s role in antiangiogenic therapy resistance in glioma. Despite the Janus-faced functions of ephrinB2–EphB4 signaling in cancer Eph signaling remains a promising clinical target.

## 1. Introduction

### 1.1. EphrinB2–EphB4 Signaling

**Figure 1:**
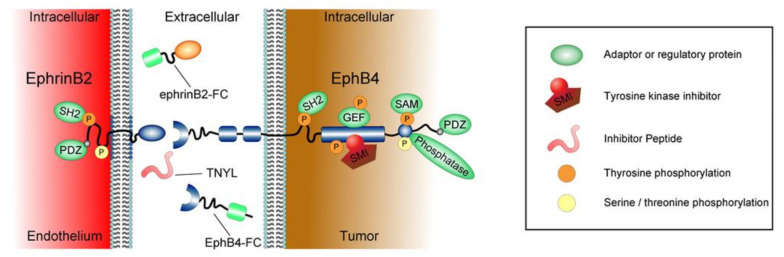
Molecular compartmentalization of ephrinB2 and EphB4. EphB4 forward signaling involves dimerization, Src homology 2 (SH2), sterile alpha motif (SAM), and PSD95, DLG, ZO1 (PDZ) autophosphorylation (orange P). Kinase independent signaling involves guanine nucleotide exchange factor (GEF) binding followed by autophosphorylation. Phosphotyrosine phosphatases are known to reduce EphB4 activity. EphrinB2 reverse signaling involves Src-mediated phosphorylation (orange P) in combination with SH2 and PDZ protein assembly. Serine residue phosphorylation was discovered in both directions (yellow P). Small molecule inhibitors (SMI) for the EphB4 kinase domain are available, the extracellular domain of ephrinB2 and EphB4 have been fused to Fragment crystallizable (ephrinB2-FC & EphB4-FC) regions to generate antibody therapies, and small interfering peptides (TNYL) are available that bind to the ligand pocket and block signaling.

The receptor tyrosine kinase (RTK) EphB4 and its ligand ephrinB2 are part of a larger group of signaling receptors, the Ephs, which have been shown to steer a multitude of physiological processes during adulthood and embryogenesis [1]. Eph receptors are involved in, among other processes, angiogenesis, vasculogenesis, cell migration, axon guidance and neurogenesis [1]. The Ephs are divided into -A and -B subclasses, depending on the way they interact with their ligands [2]. The RTKs comprise an extracellular ligand-binding domain, as well as a cysteine-rich region and two fibronectin-III repeats [3]. The intracellular part consists of the kinase as well as a sterile alpha motif (SAM) domain and a PSD95, DLG, ZO1 (PDZ)-domain (Figure 1) [4]. EphA receptors bind glycosylphosphatidyl inositol (GPI)-anchored ligands (ephrinAs), whilst EphBs bind ephrinB ligands, which feature a transmembrane domain and an intracellular PDZ-domain as well as conserved tyrosine residues. As there are uneven numbers of receptors and ligands (six EphB receptors 1–6 and three ephrinB ligands 1–3), intermingling of receptor–ligand combinations is possible and commonly observed [4,5]. Upon formation of the receptor–ligand complex, signaling is carried out bidirectionally, inside the receptor-carrying cell (“forward signaling”) and inside the ligand-carrying cell (“reverse signaling”). Forward signaling is mediated by clustering of the receptor and subsequent phosphorylation of the intracellular portions of the RTK, whilst reverse signaling is carried out similarly, by means of phosphorylation of the cytoplasmatic domains in ephrinBs (Figure 1) [4].

The molecular signaling between EphB4 and ephrinB2 is a crucial regulator of cellular interactions in a multitude of developmental and physiological processes. The underlying principal for the majority of these ligand–receptor contacts lies within the regulation of cellular repulsion and adhesion [6]. Most commonly, Eph forward signaling results in an increase in repulsive forces, whereas ephrin-based reverse signaling can have repulsive and adhesive properties, depending on cellular context and physical contact [7,8].

During development of the nervous system, Eph–ephrin signaling is crucial for cellular migration in neurons by guiding axons and mediating their formation and plasticity as well as apoptosis of neural progenitors [6,9,10,11,12] (extensively reviewed in Kania & Klein). Similarly, the growth and guidance of venous and arterial endothelial cells (ECs) is also greatly influenced by reciprocal EphB4 (on venous ECs) and ephrinB2 (on arterial ECs) expression. The complementary expression patterns help to establish clear borders between the vascular compartments [13,14]. Upon cellular-contact, the receptor- and ligand-expressing cells retract from one another through Rac-dependent endocytosis of the activated Eph receptors, thus enabling the intricate pattern development necessary for capillary network formation [15].

**Figure 2:**
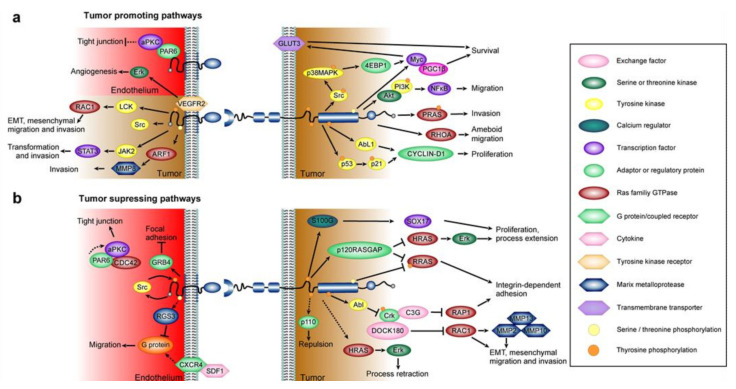
Tumor promoting and suppressing pathways identified after ephrinB2–EphB4 binding. (**a**) Tumor promoting pathways. EphrinB reverse signaling promotes invasiveness, epithelial–mesenchymal transition (EMT) and migration through STAT3, Src, RAC1 and MMP8 [47,48]. In endothelial cells, ephrinB2 reverse signaling regulates VEGFR2 internalization critical for angiogenesis [49]. Non phosphorylated ephrinB will block tight junction formation via PAR6 [50]. EphB forward signaling in tumors promotes survival via Myc and proliferation via Cyclin-D1 through multiple pathways [51,52,53]. Migration can be enhanced through the Akt/PI3K NFkB pathway [31]. Other migratory pathways of EphBs include phosphorylation of RRAS and activation of RHOA [54,55]. (**b**) Tumor suppressing pathways. Endothelial ephrinB disturbs focal adhesion via GRB4 during reverse signaling [56,57]. Tight junctions are formed after PAR6 binding and trimerization with atypical PKC (aPKC) and CDC42 [50]. Constant regulatory feedback is imposed by Src [56]. Cytokine inhibition was found trough modulation of the CXCR4R pathway [56]. Forward singling in tumors involves the upregulation of the PI3K subunit p110 [58]. Abl activation was found to inhibit RAP1 and RAC1 [20,59,60]. RRAS and HRAS are blocked after p120RASGAP activation [61]. RRAS and HRAS are also reduced by direct phosphorylation [62]. Internal calcium can be regulated by S100G which activates SOX17 and reduces proliferation [46]. Different MMPs are significantly upregulated when EphB4 is depleted [46]. Pathways identified in other celltypes but potentially active in endothelial and/or glioma cells are indicated with a dashed line.

During carcinogenesis, cancer cells can hijack these physiologic functions and subsequently increase their migratory and invasive properties [16]. A multitude of downstream signaling pathways are subsequently affected (Figure 2 and Figure 4). As mentioned above, activation of Eph receptors can induce cytoskeletal changes through Rho GTPases such as Rac1 and RhoA and in reverse signaling, Src-family kinases play a crucial role. This can occur through actively induced changes in receptor expression and phosphorylation, and is further facilitated by changes within the tumor microenvironment (TME) such as increased levels of hypoxia and VEGF signaling [17,18,19]. A comprehensive overview of the individual changes of the affected intracellular pathways is beyond the scope of this review and is extensively reviewed in [6].

It is important to bear in mind, that under pathologic conditions an increase in receptor expression does not necessarily have to correlate with increased downstream signaling, as demonstrated in a study by Noren et al [20]. Despite high levels of EphB4 expression in breast cancer cell lines, the tyrosine phosphorylation of the receptor was significantly lower than in non-transformed epithelial cells [20]. At the same time, the expression of ephrinB2 was low or undetectable in all breast cancer cell lines examined, despite the non-transformed MCF-10A human breast epithelial line [20]. This indicates that not only the expression of the receptor but also the availability and binding of ephrinB2 and the subsequent extent of kinase activation (clustering) play a pivotal role in determining pro- and antitumorigenic effects of EphB4 reverse signaling (Figure 1).

Multiple targeted drugs against ephrinB2 and EphB4 have been developed to pharmacologically manipulate this interaction in different ways (Figure 1). Some have received FDA approval, are in clinical trials, or available for translational research only (Table 1).

### 1.2. Tumor Promoting and Demoting Effects of EphrinB2–EphB4 Signaling

Seemingly opposing effects of ephrinB2–EphB4 signaling on cancer progression are found throughout the literature [37]. The overexpression of EphB4 in 22Rv1 prostate cancer and MCG-10A mammary epithelial cell lines led to an initial increase in migration, invasion and anchorage-independent growth, crucial phenotypes necessary for metastasis formation. However, in the presence of ligand-dependent signaling, these effects were reversed and EphB4 protein levels were significantly reduced [38]. Inhibition or downregulation of EphB4 was found to reduce the cancerogenic activity of multiple tumor types in vitro and in vivo. EphB4 was also found to promote the Rac/c-Raf pathway in mamma carcinoma, Akt and mTor were downstream targets of EphB4 in ovarian cancer, Integrin β8 was regulated in prostate carcinoma, and notch signaling was epigenetically upregulated by EphB4 in colorectal cancer, suggesting a pro-tumorigenic role [39,40,41,42]. Expression was high in pancreatic cancer stage III and IV compared to I and II, and a reduced malignancy was found when expression and signaling was inhibited [43]. Moreover, upregulation of EphB4 in gastric cancer and colorectal cancer resulted in increased metastasis [44,45]. In colorectal cancer, the expression of a dominant negative EphB4 was shown to facilitate growth [46]. Despite this seemingly convincing evidence for the pro-tumorigenic effects of EphB4, it is important to point out that the majority of the aforementioned studies have provided little to no information on ephrinB2 ligand expression, as their main focus was on receptor functions. As mentioned before, in this complex bidirectional signaling pathway, both ligand and receptor signaling, and activation status should be consequently addressed.

Soluble ephrin-Fc ligand has repeatedly been shown to reduce malignancy in different tumor cells in vitro and in vivo [20,37,56,59,63,64]. Hemizygous deletion of EphB4 in colon cancer induced transcriptional changes observed in the transition of adenoma-to-carcinoma phenotype [46]. In melanoma metastasis, EphB4 on tumor cells is critically involved in endothelial cell–tumor cell repulsion and downregulation or loss of ephrinB2 on endothelial cells increased bone metastatic burden in vivo [7]. Most effects are cancer type specific. For example, elimination of the ligand on endothelial cells had no influence on the metastatic behavior of Lewis lung carcinoma cells [7].

### 1.3. Endothelial EphrinB2 and EphB4 in Vasculogenesis and Angiogenesis and Antiangiogenic Therapy

EphrinB2 and EphB4 are essential proteins for the development of the cardiovascular system [13,65]. Genetic deletion of ephrinB2 and EphB4 revealed that the absence of the respective signaling molecules did not only significantly impair neuronal growth and nervous system establishment but lead to exitus in embryonic mice [66,67,68]. Analysis of the latter confirmed distorted vessel networks with severe cardiovascular defects and growth retardation [68]. Overexpression of ephrinB2 or vascular endothelial growth factor (VEGF) treatment in endothelial cells increased their sprouting activity [69]. Moreover, VEGF receptor 2 (VEGFR2) depletion also shows embryonic lethality [70,71]. VEGFR2 and ephrinB2 form critical dimers in the sprouting tips of the endothelium [49]. Absence of the ephrinB2–PDZ domain or the entire ephrinB2 molecule disturbs the internalization process essential for VEGFR2 signaling in these cells.

**Figure 3:**
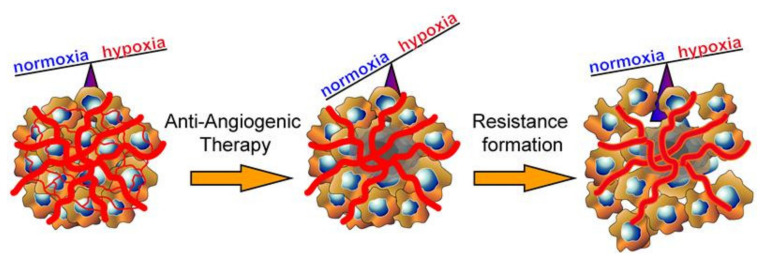
Effects of antiangiogenic therapy in glioma. Antiangiogenic treatment leads to elimination of small blood vessels followed by an increase in hypoxia and a larger necrotic tumor core. Consequently, the tumor will undergo therapy evasion and increase resistance. Phenotypically, this is manifested by increased tumor cell invasion and vascular maturation mediated by, but not limited to ephrinB2–EphB4 signaling.

Currently only Bevacizumab, a monoclonal antibody targeting VEGF, is used as a therapeutic agent for recurrent glioblastoma in the US only, based on two phase-II studies [72]. In fact, rapid resistance of the tumor vasculature is readily observed in clinical practice [73]. Several hypotheses have been proposed on why tumors develop therapeutic resistance towards antiangiogenic treatments. These include vasculature-based mechanisms such as vessel co-option, vascular intussusception and vasculogenic mimicry [74]. Vessel co-option describes the acquisition of nearby physiological vessels and inclusion into the pathological tumor vessel network by migration of tumor cells along these vessels [74]. Vascular intussusception describes pathological expansion and bifurcation of tumor vessels and vascular mimicry the incorporation of non-endothelial tumor cells to restock leaky, unfinished, or damaged tumor vessels due to antiangiogenic treatment [74]. Also increased activation of perivascular, stabilizing cells such as pericytes as well as augmented promotion of paracrine secretion of bone marrow derived cells and fibroblasts leads to an increased resistance towards antiangiogenic agents (Figure 3) [74]. It has been postulated that upon experience of hypoxic conditions following blockade of VEGF and subsequently insufficient blood flow, other proangiogenic signaling pathways are increasingly activated (Figure 3) [74,75]. These signaling pathways comprise signaling molecules such as platelet derived growth factor C (PDGF-C) of the PDGF receptor-α signaling pathway as well as basic fibroblast growth factor (bFGF), stromal-derived factor 1α (SDF-1α) and crucially, the ephrinB2–EphB4 signaling pathway [76,77]. The impact of Eph/ephrin signaling has been extensively studied in gliomas and has repeatedly been shown to promote tumor growth [78,79,80,81,82]. EphrinB2–EphB4 interaction was negatively correlated with the outcome in samples of 96 primary glioma patients [80].

## 2. EphrinB2–EphB4 in Glioma

Malignant brain tumors, derived from glial cells, such as astrocytoma or glioblastoma, represent the largest entity of non-metastatic, primary lesions of the central nervous system (CNS) [83]. These tumors represent approximately one quarter of all primary brain tumors and up to 80% of malignant primary brain tumors [83]. They are characterized by highly infiltrative and aggressive growth as well as strong vascularization [84]. Treatment options have marginally improved over the last two decades and continue to consist of maximum operative resection, followed by radiation and, depending on the degree of malignancy, chemotherapeutic treatment, mostly based on alkylating agents [84,85]. Decisive for the most malignant grade 4 glioma are classic histopathological characteristics such as necrosis and microvascular proliferation [86]. Hence, antiangiogenic treatments were initially considered to be a promising asset for this tumor entity. These therapies primarily target proangiogenic pathways and block proangiogenic factors such as VEGF [87]. Antiangiogenic treatments had already shown to be very efficient in other cancerous pathologies such as colorectal cancer, yet several phase III studies failed to show a significant effect on overall survival in malignant glioma [88,89,90,91,92]. Currently only Bevacizumab, a monoclonal antibody targeting VEGF, is used as a therapeutic agent for recurrent glioblastoma in the US only, based on two phase-II studies [72].

### 2.1. Effects of EphB4 and EphrinB2 on Angiogenesis

Interactions of EphB4 with its ligand ephrinB2 are important for vessel network formation. EphB4 is primarily expressed on venous endothelial cells (EC), while ephrinB2 is predominantly expressed by arterial ECs [93]. Interestingly, the activation of the ephrinB2–EphB4 pathway results in antagonizing effects, depending on whether forward or reverse signaling is activated [93]. Activation of forward signaling by means of truncated ephrinB2-Fcs, lacking an intracellular domain, leads to inhibition of EC migration and vascular sprouting and support of cell segregation, whereas activation of reverse signaling via EphB4-Fc effectuates the exact opposite [93]. Delicate balancing of these effects is essential for purposeful vascular network formation and prevention of non-constructive intermingling of venous and arterial ECs in the process of angiogenesis. Further influences on vascular stability have been proposed, perivascular cells such as pericytes have also been shown to express ephrinB2 [13]. As levels of both EphB4 and its ligand ephrinB2 have been found to be significantly elevated in malignant glioma, further illumination of their role in tumor angiogenesis and possible resistance towards antiangiogenics is required [94,95].

### 2.2. Effects of EphB4 and EphrinB2 on Antiangiogenic Resistance

EphrinB2–EphB4 interaction as well as their bidirectional signaling seem to exert pivotal and, at times, converse effects on progress and invasiveness of tumor cells in different tumor entities. Concerning glioma, receptor as well as ligand primarily seem to carry out tumor promoting effects by means of their respective signaling. Analysis of human glioma specimens, has shown increased mRNA expression levels of EphB4 and ephrinB2 in ECs as well as in xenograft tumor cells [94]. Also, genome profiling by means of tissue microarrays identified augmented expression of ephrinB2 in high grade human glioma cells, compared to normal brain tissue [95]. EphrinB2 gene expression eventually was proven to correlate with short term survival in malignant gliomas and its phosphorylation led to increased migration and invasion of glioma cells in vitro [95]. In addition, examination of the reverse signaling effects of ephrinB2 in glioblastoma stem-like cells, revealed mediation of cytokinesis via Rho-A signaling in vitro. Protumorigenic effects of ephrinB2-induced forward signaling are facilitated through perivascular invasion of the cells in murine glioblastoma models in vivo [96]. Knockdown of ephrinB2 in an orthotopic model of luciferase-coupled glioblastoma-like stem cells, revealed a significantly decelerated tumor growth as well as diminished contact of glioblastoma-like stem cells and endothelial cells, prohibiting vascular coupling [96]. Yet, a recent study, examined EphB4 and ephrinB2 expression in glioma in vitro, identified delineated growth suppressive effects of EphB4-signaling following phosphorylation by ephrinB2 [97]. The authors further showed spatially heterogenous expression of the two in different areas of the tumor [97]. EphB4 was only located in the tumor core, while ephrinB2 was distributed ubiquitously [97]. The spatial distribution was hypothesized to mediate the higher invasion and tumor cell migration at the rim of the tumor, compared to less malignant activity in the tumor core [97].

In summary, it appears that the balance of EphB4 and ephrinB2 expression, their respective signaling pathway activation, and special distribution influence glioma growth, development, and invasion essentially. The bidirectional signaling and receptor–ligand expression is challenging and, despite intensive research, not sufficiently understood to generalize its effects on biological behavior of malignant glioma.

Regarding specific angiogenetic effects of EphB4 and subsequent antiangiogenic resistance, endothelial overexpression of EphB4 during glioma growth in vivo, effectuates significantly enlarged tumor vessels, which seemingly organized themselves to a more structured network [94]. Further analysis showed reduced permeability of these vessels by means of an increased activation of the angiopoietin-1/Tie2 system [94]. Morphologically, these vessels resembled glioma vessels that had outlasted and proven to be resistant towards antiangiogenic treatment [98,99]. As a consequence, investigation of possible antiangiogenic resistance mechanism involving EphB4–ephrinB2, were carried out using endothelial specific EphB4 overexpression in combination with antiangiogenic treatment [77]. Glioma cell implantation and overexpression of endothelial EphB4, reduced the effect of the antiangiogenic therapy significantly [77].

Live imaging of the tumor vessels, in two modalities (chronic cranial window and dorsal skinfold chamber), revealed large, well perfused EphB4 tumor vessels [77]. EphB4 expression in endothelial cell reduced apoptosis, sustained proliferation, and sustained pericyte–endothelial cell interaction [77]. The exact molecular mechanism forming these resistant blood vessels, remains a topic of further investigation. We hypothesize, that EphB4 forward signaling was responsible for the depicted results, as the Eph receptor outnumbered the ephrinB2 ligand in these experiments.

A possible molecular pathway, that involves ephrinB2–EphB4 mediated therapeutic resistance, is the Notch signaling pathway, which is equally relevant for viability of embryonic mice [100]. Activation of the Notch receptor through its ligand DLL4, leads to proteolytic cleavages of the receptor, effectuating several downstream pathways and results in increased transcription of ephrinB2 [101]. DLL4 expression increases in higher graded glioma and correlates with the maturation status of tumor vessels [102,103].

**Figure 4:**
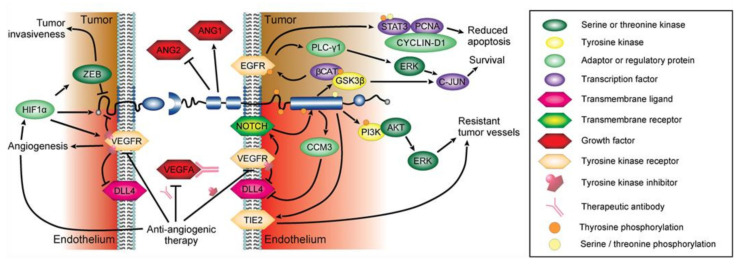
EphB4 resistance mechanisms towards antiangiogenic treatment in glioma. EphrinB2 expression in tumors is reduced after antiangiogenic treatment, arbitrated by means of elevated levels of hypoxia-inducible factor (HIF-1α) and zinc finger E-box-binding homeobox 2 (ZEB2) [107]. Among other effects, this pathway enhances tumor invasiveness. In forward signaling, EphB4 controls the activation of the EGF receptor via the Wnt/beta-catenin signaling pathway. The EGF receptor phosphorylates phosphoinositide phospholipase C-gamma-1 (PLC-γ1), which was found to drive gene expression changes in Sunitinib resistant glioma [105]. PLC-γ1 phosphorylation increases tumor invasiveness in other tumor entities. In the endothe-lium, antiangiogenic therapy blocks VEGFR–EphrinB2 dimerization and Delta-Like Canonical Notch Ligand 4 (DLL4) activa-tion [106]. EphB4 is progressively expressed in Bevacizumab resistant blood vessels and regulates DLL4/Notch proportions via cerebral cavernous malformation 3 (CCM3) [103,106]. Endothelial EphB4 overexpression forms large, treatment insensitive vessels expressing increased amounts of TIE2 and ANG1 and decreased amounts of ANG2 [94]. Additionally, it protects cells from apoptosis possibly through the PI3K-AKT-ERK pathway [106].

DLL4-overexpressing human glioblastoma cell lines, treated with Bevacizumab, displayed a clear antiangiogenic effect of DLL4 towards the VEGF receptor inhibitor [103]. Inhibition of the DLL4 overexpression reversed this effect; immunohistochemical staining of the treatment resistant tumors revealed large, treatment-insensitive vessels, similar to the ones of the earlier described in EphB4-overexpressing vessels [77]. Moreover, EphB4 expression increases in DLL4-overexpressing tumors [103]. Anti-ephrinB2 antibody or soluble EphB4 protein inhibition, in combination with Bevacizumab, abrogated the resistant effect of DLL4-overexpressing tumors (Figure 4) [103].

Alternatively, EGF receptor signaling pathway activation via EphB4 were postulated after parallel increased levels of EphB4 and epidermal growth factor (EGF) receptor were discovered [104]. EGF signaling augments PLC-*γ*1 phosphorylation, a critical downstream effector mediating tumor invasiveness and resistance towards antiangiogenic treatments such as Sunitinib (Figure 4) [105]. Additionally, activation of PI3K-AKT and ERK will promote cell survival downstream of EphB4 [106].

Moreover, ephrinB2 takes part in antiangiogenic resistance in malignant glioma. Depner et al. revealed, that ephrinB2 was deactivated by zinc finger E-box-binding homeobox 2 (ZEB2), a transcription factor of the transforming growth factor *β*- signaling pathway, following activation by the hypoxia-inducible factor (HIF)-1α [107]. The authors further showed, that ZEB2 and HIF-1α were upregulated in antiangiogenic therapy resistant glioblastoma cells, while ephrinB2 expression was diminished (Figure 4) [107]. Deactivation of ephrinB2 effectuated a remarkably increased invasiveness of the glioma cells, while depletion of ZEB2 reversed this effect [107]. These results have been partially confirmed in a recent study, where decelerated tumor growth in glioma, followed a predominant activation of ephrinB2-mediated reverse signaling [108]. In contrast, augmented activation of EphB4 forward signaling promoted tumor progress [108]. However, it has to be considered, that ZEB2 and HIF-1α themselves have been described to promote tumor invasiveness, progression and, epithelial–mesenchymal transition in cancer, wherefore the antiangiogenic influence of ephrinB2 remains notional [109,110].

In summary, it is not possible to dismiss the antiangiogenic effects of EphB4 and ephrinB2 in terms of mediating resistance towards antiangiogenic treatment in glioma. The receptor and its ligand exert antithetical effects on tumor growth and mediation of resistance. EphB4 by means of overexpression, whereas ephrinB2 exerts this role by downregulation of the ligand or diminished activation. Possible intracellular pathways include the DLL4 signaling pathway, the PI3K signaling pathway and, the EGF receptor pathway for EphB4 forward signaling and the HIF-1*α*–ZEB2 pathway for ephrinB2 reverse signaling (Figure 4). Several interceptors of these signaling pathways in combination with antiangiogenic treatment have been used successfully to overcome the activation of the drug evasive effects of EphB4 and ephrinB2 [107,108].

## 3. EphrinB2–EphB4 in Neurooncological Metastasis

### 3.1. Effects of EphrinB2–EphB4 on Brain Metastasis

Brain metastases (BM) are the most common brain tumors in adults, with a steadily increasing incidence, currently approximated at 200,000 patients per year in the US alone [111,112]. Despite recent advances in systemic and local treatment options, median survival is still poor and has only slightly improved over the past decades [113]. EphB receptors are overexpressed in a multitude of human cancers and overexpression has repeatedly been correlated with a more aggressive disease phenotype [114,115,116]. Among the most consistently upregulated EphB receptors is EphB4, which—similar to its ligand, ephrinB2—has consistently been detected in a wide variety of human tumor cell lines [117,118]. For a more extensive review of this topic, please see Pergaris et al. in this current IJMS issue [118].

Bearing in mind that brain metastases are more commonly diagnosed than primary gliomas described before, the extent of research into Eph–ephrin signaling has unfortunately not been as extensive and pre-clinical research is lacking. EphrinB1 has been identified as a potential therapeutic target after proteotranscriptomic profiling of a brain metastatic breast cancer cell line (MDA-MB-231) [119]. The authors of this study suggested a possible association with HER2 expression and identified ephrinB1 as a potentially targetable molecule to reduce breast cancer BM [119]. Furthermore, EphA2 expression in a retrospective immunohistochemical analysis of 264 NSCLC tumors correlated with survival and predicted brain metastases [120]. Nevertheless, ephrinB2 and EphB4 signaling has, to the best of our knowledge, not been studied in the setting of brain metastases.

### 3.2. Effects of EphrinB2–EphB4 on Spinal Bone Metastasis

According to recent estimates, around 70% of cancer patients will develop distant metastases during the course of their disease and another 70% of metastatic cancer patients are affected by spinal bone metastases, making it one of the most common sites of cancer spread [121]. The most common tumors that spread to the spinal bones are primary breast, prostate and lung cancer [122]. The majority of spinal metastases are located in the thoracic spine (~70%), followed by lumbar (~20%) and cervical spine (~10%). In over 50% of the cases, multiple levels of the spinal cord are simultaneously affected [123]. Osseous metastases of the spine are often diagnosed at an advanced disease stage, and can subsequently lead to rapidly progressive epidural compression with significant effect on ambulatory status and sensory and motor functions [124].

The current standard of care consists of immediate surgical decompression of the spinal cord, accompanied by reconstructive and stabilizing procedures if necessary, followed by radiotherapy of the metastatic site [125]. Yet, despite recent advances in treatment, the median survival after metastatic epidural spinal cord compression (MESCC) remains low and is calculated in months rather than years. Main factors affecting median survival are tumor type, ambulatory status, time to development of metastasis as well as number and site of spinal lesions [126].

The balance between resorption and formation of osseous structures through tightly regulated cellular crosstalk is disrupted in malignant diseases, and leads to the formation of osteoblastic (typically observed in metastatic prostate cancer) and osteolytic (typically observed in metastatic breast cancer and multiple myeloma) metastatic lesions [127]. Bidirectional ephrin-B2–EphB4 signaling plays a crucial role in preserving the delicate balance between bone-resorbing osteoclasts and bone-forming osteoblasts [16,69]. It was found that ephrinB2 is expressed on osteoclasts, whereas EphB4, along with other Eph family members, was initially described on osteoblasts. EphrinB2 reverse signaling suppressed osteoclast differentiation through a negative feedback that inhibited c-Fos and NFATc1, while EphB4 forward signaling increased osteogenic differentiation of osteoblasts and was able to induce bone mass in a model of experimental overexpression [69]. Besides osteoclasts, ephrinB2 is present on the surface of osteoblasts as well. Cell-specific ablation significantly delayed osteoblast differentiation and increased apoptosis, suggesting that ephrinB2 helps maintaining the osteoblast lineage through antiapoptotic effects [128,129]. Furthermore, ephrinB2 expression is stimulated by parathyroid hormone (PTH) and its related peptide (PTHrP). PTH signaling modified by EphB4 mediates insulin-like growth factor-1 (IGF-1) activity, proving a crucial involvement of Eph–ephrin signaling with two of the most important pathways in bone formation [130,131]. In pathological settings, myeloma cells downregulate EphB4 expression in osteoblasts, thus decreasing bone formation and strengthening the hypothesis that Eph–ephrin signaling has significant effects on bone metastasis formation [132].

**Figure 5:**
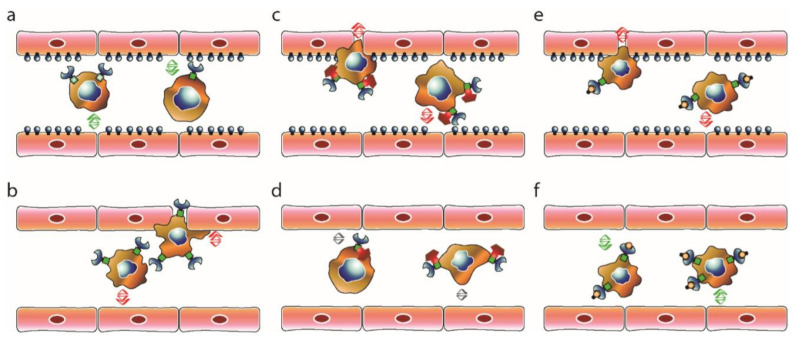
Effects of ephrin-B2–EphB4 interaction on extravasation of circulating melanoma cells in the spine. (**a**) Physi-ological interaction between EphB4 expressing melanoma cells and ephrin-B2 expressing spinal endothelial cells leads to re-pulsion (green arrows) of CTCs and hampers extravasation. (**b**) Conditional KO of ephrin-B2 on spinal endothelium decreases repulsive forces and enables CTC extravasation (red arrows). (**c**) Increased extravasation is also observed under small mole-cule inhibition (NVP-BHG 712) of EphB4 forward signaling. (**d**) No significant effects (grey arrows) on CTC extravasation in absence of endothelial ephrin-B2. (**e**) Binding of soluble ephrin-B2-Fc to EphB4 in presence of endothelial Ephrin-B2 decreases CTC repulsion and enables extravasation. (**f**) In the absence of endothelial ephrin-B2, soluble ephrin-B2-Fc partially restores physiologic barrier functions and decreases CTC extravasation.

In an initial push to explore this signaling in metastatic disease we found that ephrinB2-mediated EphB4 activation is crucial for repulsion of circulating melanoma cells from the spinal bone endothelium (Figure 5a) [7], despite previous evidence that the bone endothelium is a passive recipient of microbeads and cells alike [133]. We therefore hypothesized that, similar to the repulsive effects observed between the EphB4- and ephrin-B2-expressing endothelial cells during vasculogenesis, an analogous molecular mechanism acts as a “natural barrier” of extravasation in the spinal endothelium, enabling the repulsion of circulating tumor cells (CTCs) and thus hindering extravasation and metastasis formation. Indeed, inhibition of the physiological tumor-endothelial ephrinB2–EphB4 interaction led to increased spinal bone metastasis, earlier appearance of hind-limb locomotion deficits and shorter survival in a systemic melanoma metastasis model. This effect was achieved by selective ephrinB2 depletion on endothelial cells (Figure 5b) as well as direct small molecule inhibition (SMI) of EphB4 expressed on the CTC surface (Figure 5c) [7].

### 3.3. Therapeutic Intervention of EphrinB2–EphB4 Neurooncological Metastasis

The widespread expression of Eph–ephrin on distinct cancer types as well as its major functions in crucial steps of carcinogenesis, suggest significant therapeutic opportunities. Our group recently evaluated the effects of pharmacological EphB4 stimulation and inhibition in our spinal metastatic melanoma model [7,134]. Both ligand-dependent and –independent contexts, as well as preventative and post-diagnostic approaches were evaluated to shed light on the complex molecular interaction underlying early and late steps of spinal melanoma metastasis. Intriguingly, contradictory to our initial expectations, additional EphB4 stimulation with soluble ephrinB2-Fc during metastatic cell seeding led to an increase in spinal metastases in the presence of endothelial ephrinB2 (Figure 5e). However, the opposite effect was achieved in absence of spinal endothelial ephrinB2 (Figure 5f), again underlining the necessity to take all aspects of the molecular interaction into consideration [134]. Blocking the EphB4 kinase activity by application of the small molecule inhibitor NVP-BHG 712 did not significantly affect the number of spinal metastases or time until neurologic deficit in absence of endothelial ephrinB2 (Figure 5d). Though seemingly contradictory with the notion that EphB4–ephrinB2 interaction reduces spinal metastasis formation through CTC repulsion, these results indicate that both forward and reverse signaling pathways are necessary to achieve sufficient protection from extravasation and strengthen the notion that both receptor and ligand availability need to be incorporated in future analyses of Eph–ephrin signaling in metastatic disease. While the binding of EphB4 by soluble ephrinB2-Fc may lead to an increase in EphB4 phosphorylation in a model lacking endothelial ephrinB2, the lack of reverse signaling of the ephrinB2-expressing endothelial cells cannot recreate the entirety of the molecular interactions. The importance of ligand signaling availability was also demonstrated in the post-diagnostic setting, in which EphB4 kinase inhibition increased metastasis growth solely in presence of endothelial ephrinB2 [134].

## 4. Conclusions & Perspectives

We start to understand how tissue morphogenesis is regulated in a cell–cell contact-dependent manner. The ephrins and their receptors (Ephs) are major players involved in this signaling. Tumors are hijacking these systems to gain access to new territory and form new blood vessels. Here we described the importance of ephrinB2 and EphB4 for neurooncological diseases in particular. They are involved in antiangiogenic resistance in glioma and are critical for extravasation of metastatic melanoma cells into the spinal bone. However, clinical translation is currently still difficult, and it is important to point out, that a lot of the studies reviewed here included experiments that were performed in different settings (in vivo vs. in vitro), as well as in different species. Moreover, detailed research of the bidirectional signaling pathways through individual and simultaneous receptor/ligand stimulations in combination with bilateral cell observations remain mostly unaccounted for. Still a positive perspective for the future resembles that multiple multi kinase inhibitors (e.g., Dasatinib, Imatinib and Bosutinib) are now in clinical phase-IV studies (approved), although how much of their effectiveness in patients is caused by the inhibition of ephrinB2–EphB4 is unclear. Moreover, most of these inhibitors target the EphB4 kinase domain even though non-kinase dependent signaling and reverse signaling are induced after ephrinB2 binding. This problem is solved by a second generation of peptide inhibitors based on TNYL-RAW, targeted substitution of amino acids resulted in the BIDEN-AP peptide that was found to inhibit both forward and reverse signaling alike [135]. From the basic research standpoint, we start to learn that non-sprouting angiogenesis exists and conventional drugs are largely ineffective in blocking these pathologies [136]. VEGF is a large contributor to the angiogenesis phenotype found in tumors. However, the strategy of solely targeting one agent with one function in the highly adaptive tumor environment seems inadequate, the best efficacy is to be expected from combination treatments with additional therapeutics. The bidirectional ephrinB2–EphB4 signaling pathway harbors great potential in this regard, as shown by multiple promising pre-clinical trials outlined in this review. Further and deeper investigation of intracellular signaling pathways, the complex cellular and molecular interactions, and investigation of -omic data will help identify novel links to delineate the pro- or antitumorigenic roles of the signaling. Given the multiple roles of ephrinB2 and EphB4 in tumor growth, translational progress is desperately needed and could open new perspectives for cancer patients.

## Figures and Tables

**Table 1:** Small molecules and antibodies targeting ephrinB2 and EphB4.

Drug	Target	Clinical Phase	Binding Domain	Drug Type	IC_50_
Dasatinib	EphB4, EphA2, EphA4	FDA approved failed in glioma [21] failed in metastasis [22]	Kinase domain	Kinase inhibitor	17 nM [23], 5.5 nM [24]
Imatinib	EphB4	FDA approved failed in glioma [25] possible adverse effects in metastasis [26]	Kinase domain	Kinase inhibitor	5% at 5 × 10^3^ nM [27]
Bosutinib	EphB4	FDA approved failed in glioma [28] potentially beneficial in metastasis [29]		Small molecule	5.5 nM [27]
JI-101	EphB4	Phase 2		Small molecule	not reported [30]
XL647	EphB4	Phase 2	Kinase domain	Small molecule	1.4 nM [31]
NVP-BHG712	EphB4, EphA2	Research only	Kinase domain	Small molecule	3 nM [32]
TNYL-RAW	EphB4	Research only	Ligand binding pocket	Peptide	15 nM [33]
ephrinB2-FC	EphB4	Research only	Ligand	Monoclonal antibody	0.3 μM [34], 9 nM [35]
EphB4-FC	ephrinB2	Research only	Ligand	Monoclonal antibody	0.3 μM [36]
